# Lt. Martin F. Nolan

**Published:** 1918-12

**Authors:** 


### OBITUARY

**Lt. Martin F. Nolan,** Buffalo 1912, of N. Tonawanda, died in the military service in France, Oct. 8, of pneumonia, aged 32. He went overseas in July 1917. He had been exposed to pneumonia in line of duty. He was a brother of Brig. Gen. Dennis Nolan and Lt. Col. Daniel Nolan.

